# Supplementary material for: Structure and engineering of Brevibacillus laterosporus Cas9
Source: Commun Biol. 2024 Jul 3;7:803. doi: 10.1038/s42003-024-06422-z (PMC11222456; doi:10.1038/s42003-024-06422-z)
Supplement: Supplementary file 2 — Supplementary Information [file 42003_2024_6422_MOESM2_ESM.pdf]

Supplementary Information for

Structure and engineering of *Brevibacillus laterosporus* Cas9

Toshihiro Nakane[1,10], Ryoya Nakagawa[1,10], Soh Ishiguro[2], Sae Okazaki[3], Hideto Mori[4,5,6], Yutaro Shuto[1], Keitaro Yamashita[3], Nozomu Yachie[2,6,7], Hiroshi Nishimasu[3,8,9]*, and Osamu Nureki[1]*

*Correspondence: nisimasu@g.ecc.u-tokyo.ac.jp, nureki@bs.s.u-tokyo.ac.jp

This PDF file includes:

Supplementary Figures 1 to 6

Supplementary Tables 1 to 4

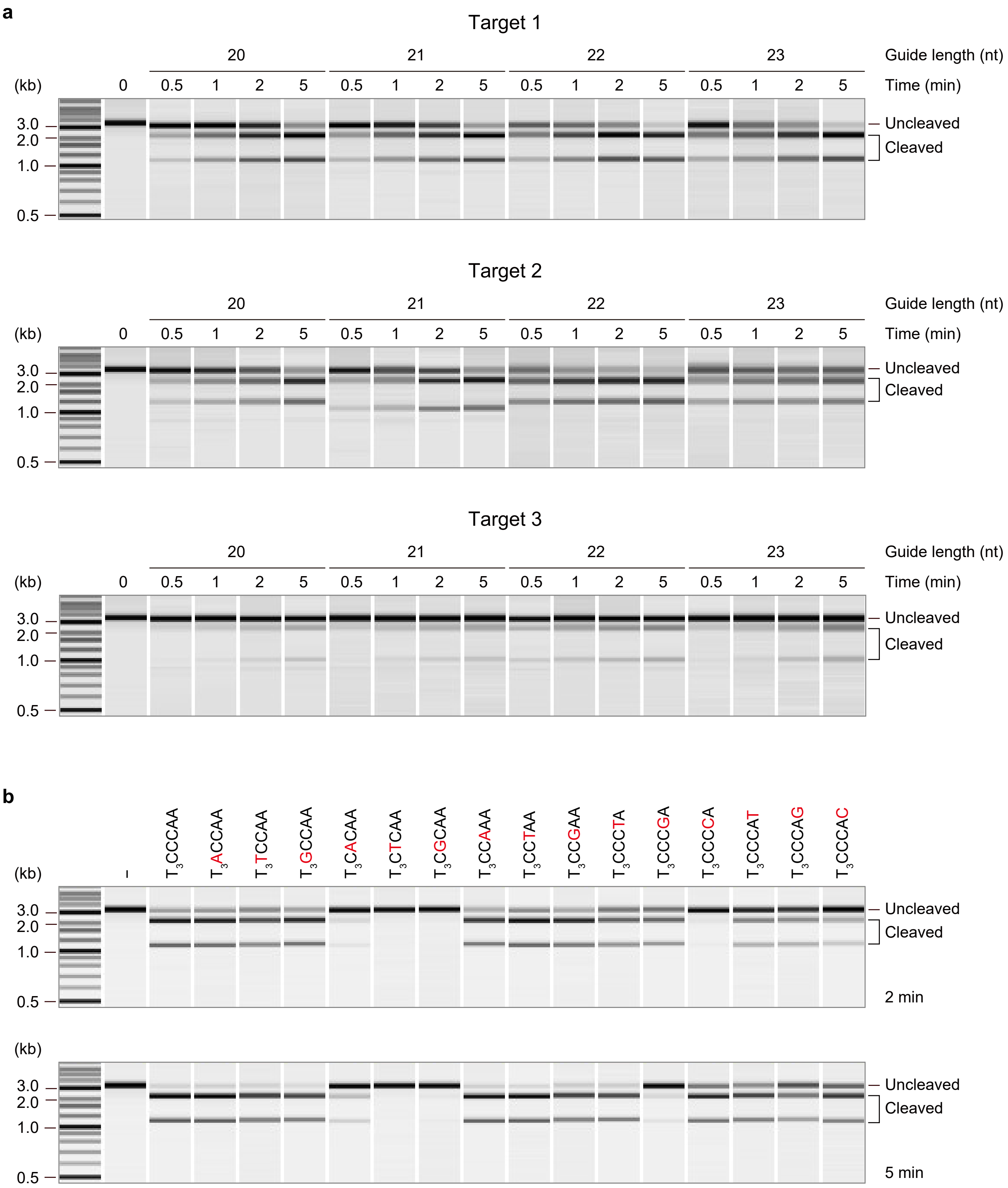

Supplementary Fig. 1 | *In vitro* DNA cleavage activities of BlCas9 toward different PAMs.
(**a**) *In vitro* DNA cleavage activities of BlCas9 with the 20–23-nt guide sgRNAs toward three different target sequences (Targets 1–3). The linearized plasmid target bearing the T_3CCCAAA (Target 1) or T_3CCCAAA (Targets 2 and 3) PAM was incubated with the BlCas9–sgRNA complex at 37°C for 0.5, 1, 2, and 5 min. The cleavage products were then analyzed by a MultiNA microchip electrophoresis system.
(**b**) *In vitro* DNA cleavage activities of BlCas9 toward DNA targets (Target 1) with different PAMs. The linearized plasmid targets were incubated with the BlCas9–sgRNA complex at 37°C for 2 and 5 min. The cleavage products were then analyzed by a MultiNA microchip electrophoresis system.

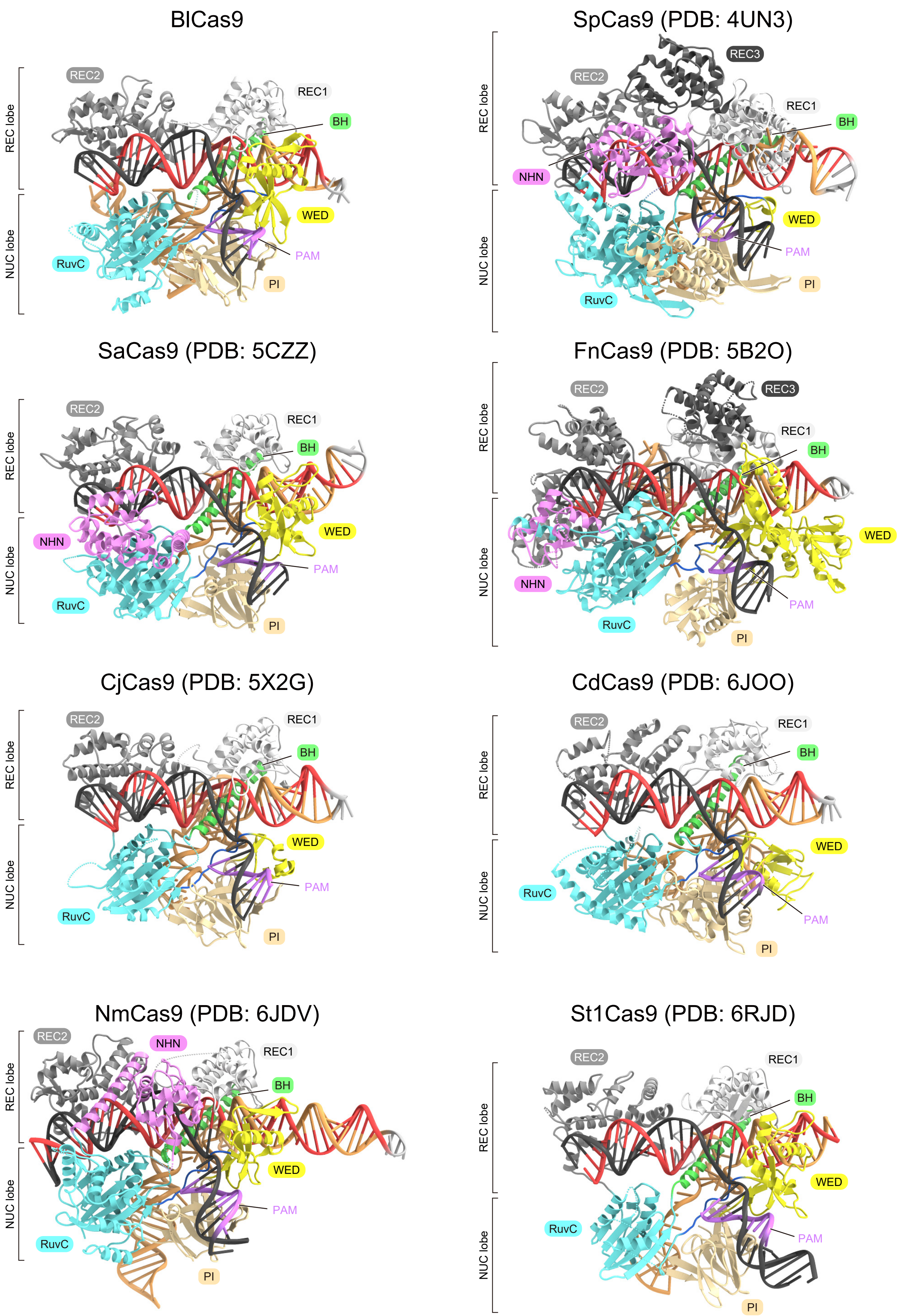
BlCas9
REC2
REC1
BH
WED
PAM
PI
RuvC
REC lobe
NUC lobe
SpCas9 (PDB: 4UN3)
REC3
REC2
REC1
BH
NHN
WED
PAM
PI
RuvC
REC lobe
NUC lobe
SaCas9 (PDB: 5CZZ)
REC2
REC1
BH
NHN
WED
PAM
RuvC
PI
REC lobe
NUC lobe
FnCas9 (PDB: 5B2O)
REC2
REC3
REC1
BH
WED
NHN
PAM
RuvC
PI
REC lobe
NUC lobe
CjCas9 (PDB: 5X2G)
REC2
REC1
BH
WED
PAM
RuvC
PI
REC lobe
NUC lobe
CdCas9 (PDB: 6JOO)
REC2
REC1
BH
WED
RuvC
PAM
PI
REC lobe
NUC lobe
NmCas9 (PDB: 6JDV)
REC2
NHN
REC1
BH
WED
PAM
RuvC
PI
REC lobe
NUC lobe
St1Cas9 (PDB: 6RJD)
REC2
REC1
BH
WED
RuvC
PAM
PI
REC lobe
NUC lobe

Supplementary Fig. 2 | Structural comparison of the Cas9 orthologs.
Structural comparison of BlCas9 with SpCas9 (Cas9 from *Streptococcus pyogenes*) (PDB: 4UN3), SaCas9 (Cas9 from *Staphylococcus aureus*) (PDB: 5CZZ), FnCas9 (Cas9 from *Francisella novicida*) (PDB: 5B2O), CjCas9 (Cas9 from *Campylobacter jejuni*) (PDB: 5X2G), CdCas9 (Cas9 from *Coryne-bacterium diphtheriae*) (PDB: 6JOO), NmCas9 (Cas9 from *Neisseria meningitidis*) (PDB: 6JDV), and St1Cas9 (Cas9 from *Streptococcus thermophilus*) (PDB: 6RJD). These Cas9 orthologs commonly adopt bilobed architectures, consisting of REC and NUC lobes, with the guide RNA–target DNA heteroduplex accommodated within the central channel. The REC lobe mainly consists of α helices, whereas the NUC lobe consists of the RuvC, HNH, WED, and PI domains. Disordered regions are indicated by dotted lines.

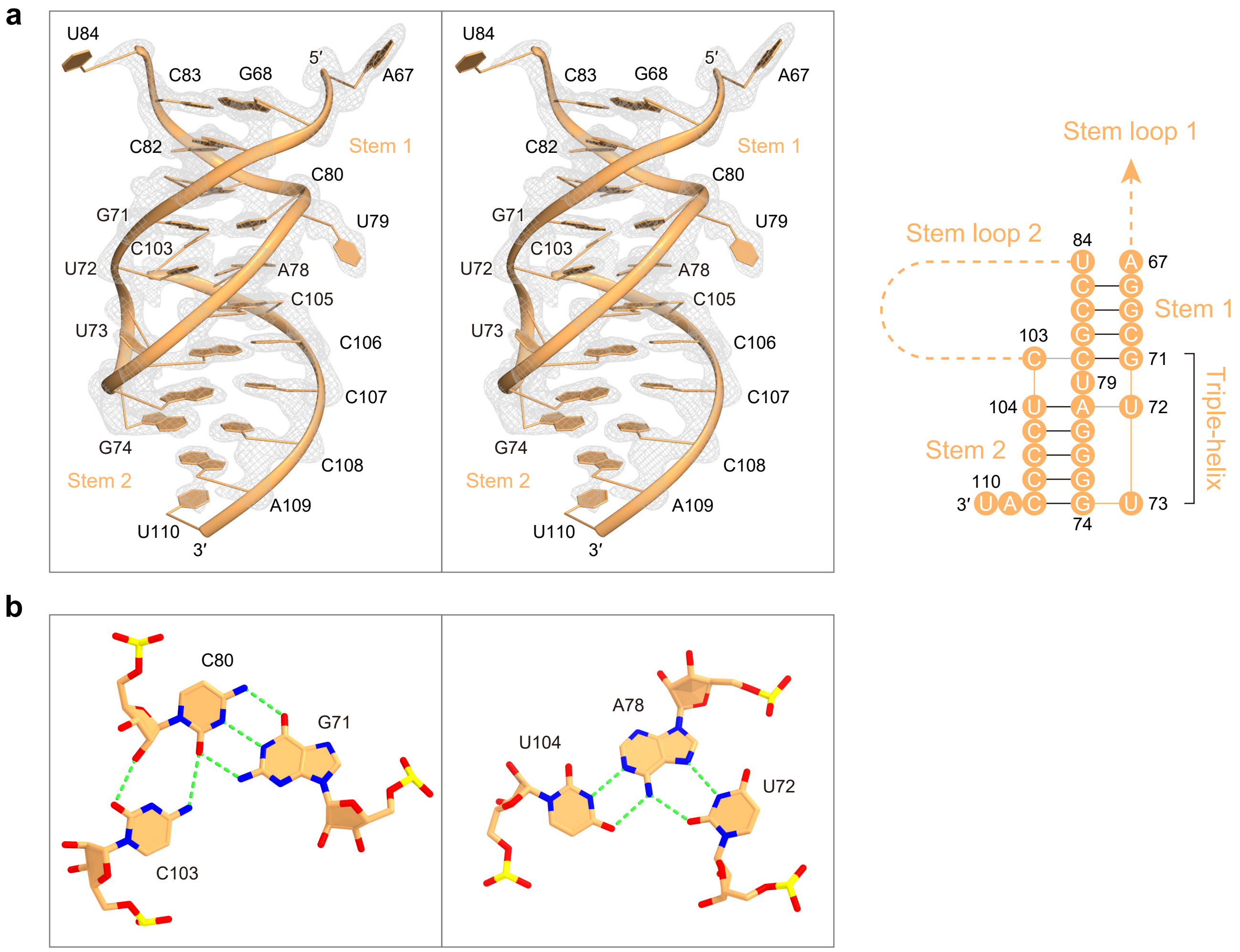

Supplementary Fig. 3 | Triple-helix structure.
(**a**) *2mFo* − *DFc* electron density map for the triple-helix structure (contoured at 2.0σ) (stereo view).
(**b**) G71:C80-C103 (left) and U72-A78:U104 (right) base triples in the triple-helix structure.

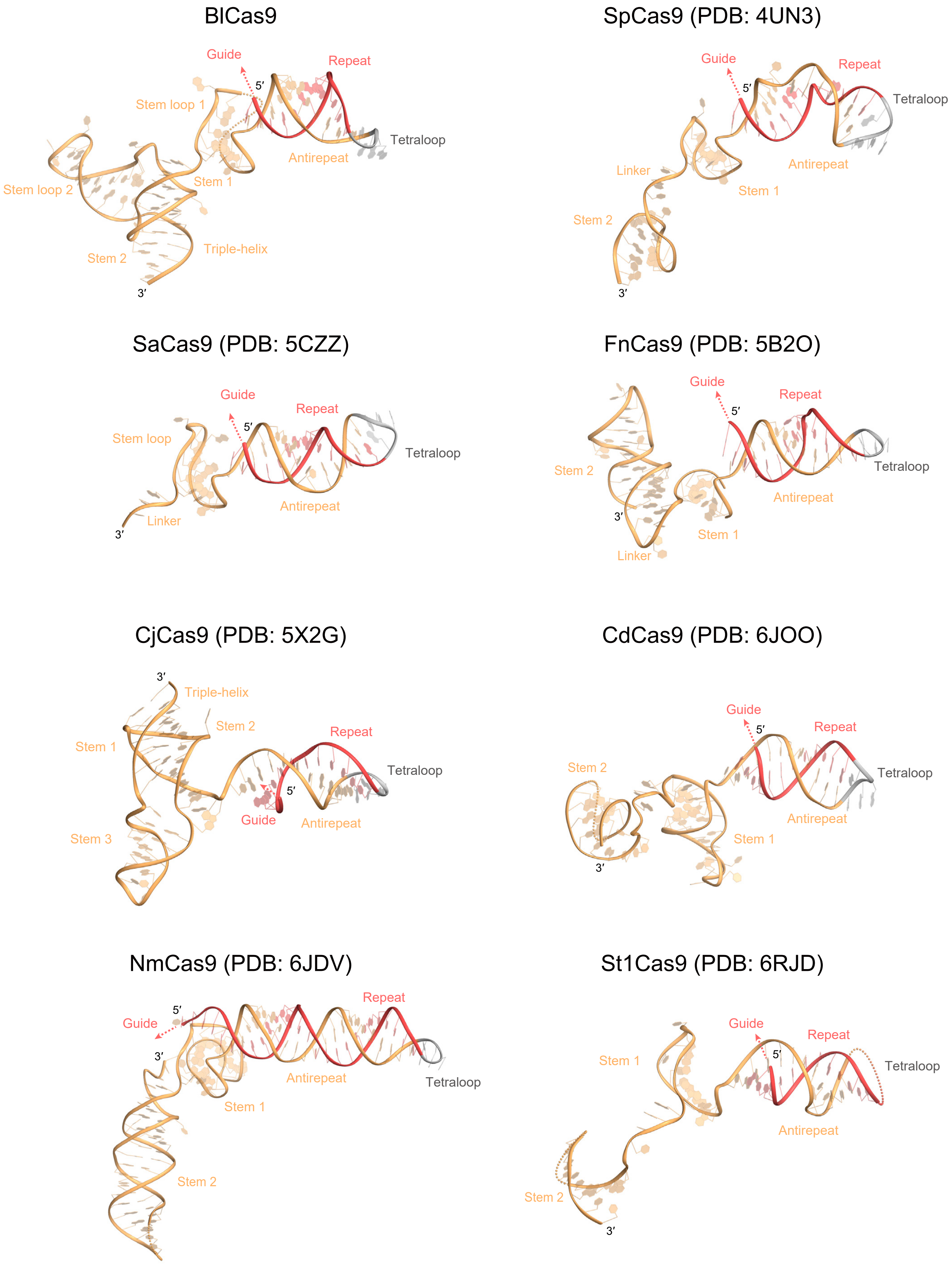

Supplementary Fig. 4 | Structural comparison of guide RNAs of different Cas9 orthologs. Structural comparison of the BlCas9 sgRNA with the sgRNAs of SpCas9 (PDB: 4UN3), SaCas9 (PDB: 5CZZ), FnCas9 (PDB: 5B2O), CjCas9 (PDB: 5X2G), CdCas9 (PDB: 6JOO), NmCas9 (PDB: 6JDV), and St1Cas9 (PDB: 6RJD). The sgRNAs commonly contain a repeat:antirepeat duplex, while the other tracrRNA scaffolds have highly diverse sequences and structures.

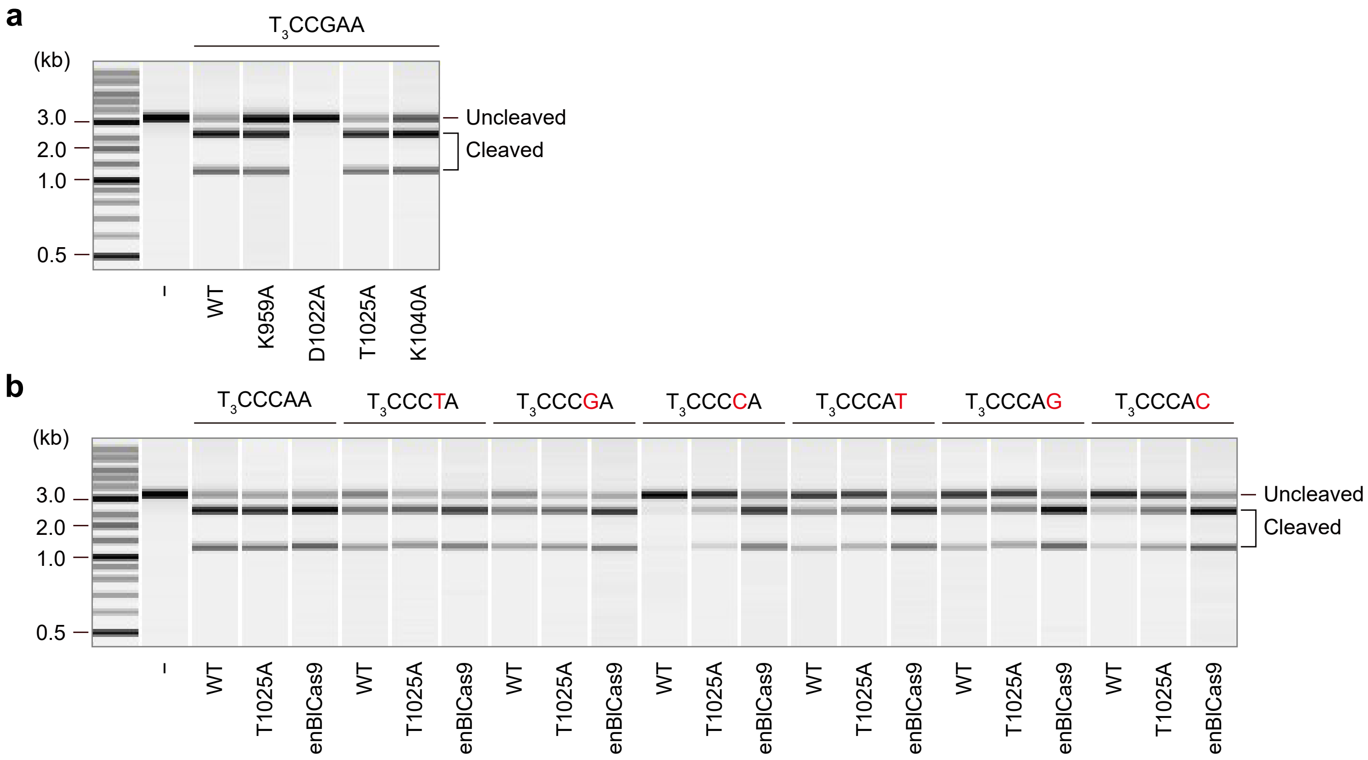

Supplementary Fig. 5 | *In vitro* DNA cleavage activities of the PAM recognition mutants.
(**a**) *In vitro* DNA cleavage activities of the PAM recognition mutants toward DNA targets with the T_3CCGAA PAM. The linearized plasmid targets were incubated with the BlCas9–sgRNA complex at 37°C for 2 min, and the cleavage products were then analyzed by a MultiNA microchip electrophoresis system.
(**b**) *In vitro* DNA cleavage activities of BlCas9 (WT), the T1025A variant, and the enBlCas9 (E904R/T1025A) variant toward DNA targets with different PAMs. The linearized plasmid targets were incubated with the BlCas9–sgRNA complex at 37°C for 2 min, and the cleavage products were then analyzed by a MultiNA microchip electrophoresis system.

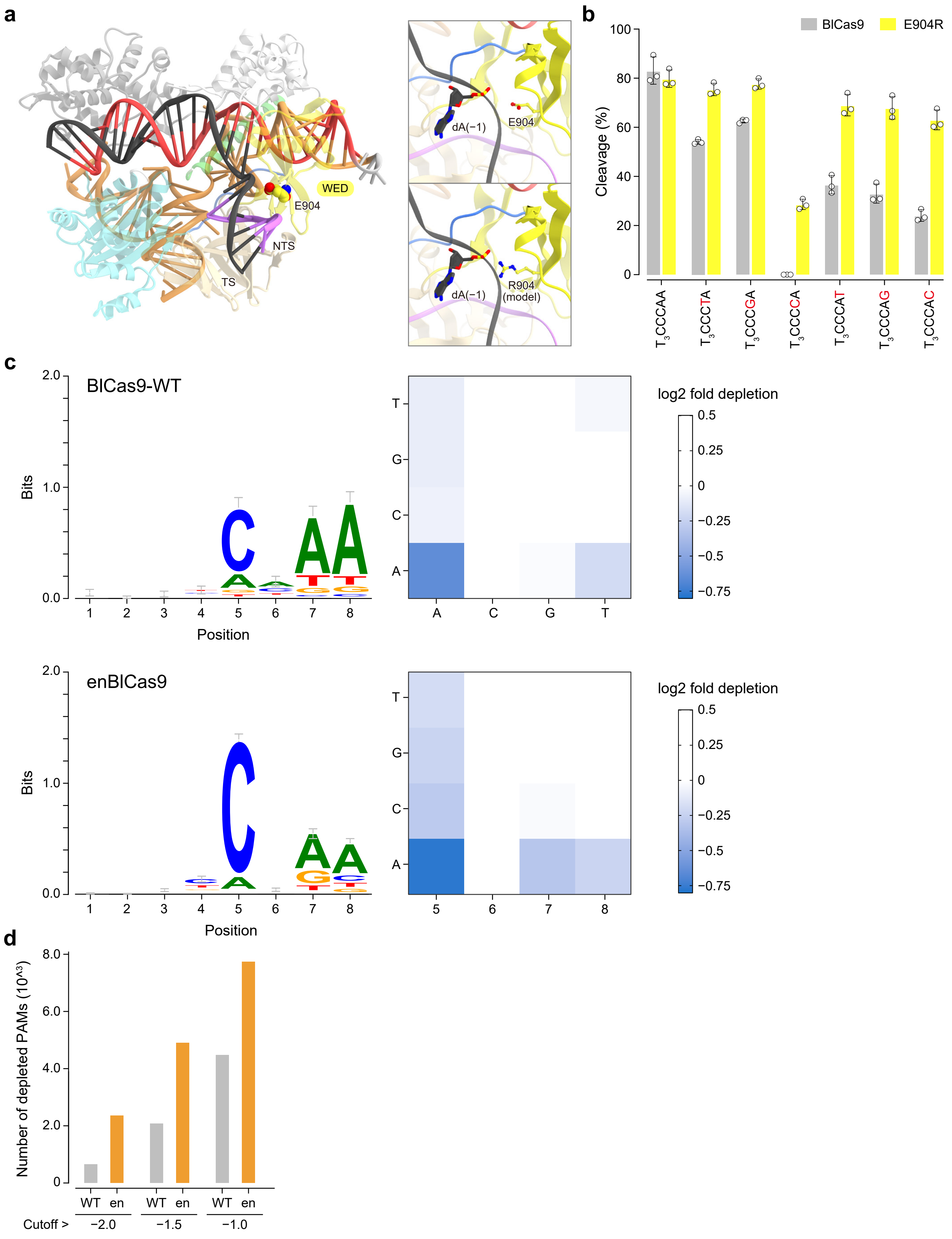
a
WED
E904
NTS
TS
dA(−1)
E904
dA(−1)
R904 (model)
b
BlCas9
E904R
Cleavage (%)
100
80
60
40
20
0
T3CCCAA
T3CCCTA
T3CCCGA
T3CCCCA
T3CCCAT
T3CCCAG
T3CCCAC
c
BlCas9-WT
Bits
2.0
1.0
0.0
1 2 3 4 5 6 7 8
Position
T
G
C
A
A C G T
log2 fold depletion
0.5
0.25
0
−0.25
−0.5
−0.75
enBlCas9
Bits
2.0
1.0
0.0
1 2 3 4 5 6 7 8
Position
T
G
C
A
5 6 7 8
log2 fold depletion
0.5
0.25
0
−0.25
−0.5
−0.75
d
Number of depleted PAMs (10^3)
8.0
6.0
4.0
2.0
0
WT en WT en WT en
Cutoff >
−2.0
−1.5
−1.0

Supplementary Fig. 6 | Molecular engineering of BlCas9.
(**a**) Mapping of the E904R mutation onto the BlCas9–sgRNA–target DNA complex. Glu904 in the WED domain is located close to the PAM duplex. Modeling suggested that Arg904 interacts with the phosphate backbone of dA(−1).
(**b**) *In vitro* DNA cleavage activities of BlCas9 (WT) and the E904R variant toward DNA targets with different PAMs. The linearized plasmid targets were incubated with the BlCas9–sgRNA complex at 37°C for 2 min, and the cleavage products were then analyzed by a MultiNA microchip electrophoresis system. Data are mean ± s.d. (n = 3).
(**c**) Sequence logos and 2D profiles of BlCas9 (WT) and enBlCas9, obtained from the PAM identification assay.
(**d**) Number of depleted PAMs. A comparison of the numbers of depleted PAMs indicates that enBlCas9 exhibits relaxed PAM recognition. en, enBlCas9.

Supplementary Table 1. sgRNA and target DNA sequences used for *in vitro* cleavage assay.

Name	Sequence
sgRNA20_ Target 1	GGAAAUUAGGUGCGCUUGGCGCUAUAGUUCCUUGAAAAAGUUGCUAUAGUAAGGGC AACAGACCCGAGGCGUUGGGGAUCGCCUAGCCCGUUUUUACGGGCUCUCCCCAU
sgRNA21_ Target 1	GGGAAAUUAGGUGCGCUUGGCGCUAUAGUUCCUUGAAAAAGUUGCUAUAGUAAGGG CAACAGACCCGAGGCGUUGGGGAUCGCCUAGCCCGUUUUUACGGGCUCUCCCCAU
sgRNA22_ Target 1	GGGGAAAUUAGGUGCGCUUGGCGCUAUAGUUCCUUGAAAAAGUUGCUAUAGUAAGG GCAACAGACCCGAGGCGUUGGGGAUCGCCUAGCCCGUUUUUACGGGCUCUCCCCAU
sgRNA23_ Target 1	GGGGGAAAUUAGGUGCGCUUGGCGCUAUAGUUCCUUGAAAAAGUUGCUAUAGUAAG GGCAACAGACCCGAGGCGUUGGGGAUCGCCUAGCCCGUUUUUACGGGCUCUCCCCAU
sgRNA20_ Target 2	GAGUCAGGCAACUAUGGAUGGCUAUAGUUCCUUGAAAAAGUUGCUAUAGUAAGGGC AACAGACCCGAGGCGUUGGGGAUCGCCUAGCCCGUUUUUACGGGCUCUCCCCAU
sgRNA21_ Target 2	GGAGUCAGGCAACUAUGGAUGGCUAUAGUUCCUUGAAAAAGUUGCUAUAGUAAGGG CAACAGACCCGAGGCGUUGGGGAUCGCCUAGCCCGUUUUUACGGGCUCUCCCCAU
sgRNA22_ Target 2	GGGAGUCAGGCAACUAUGGAUGGCUAUAGUUCCUUGAAAAAGUUGCUAUAGUAAGG GCAACAGACCCGAGGCGUUGGGGAUCGCCUAGCCCGUUUUUACGGGCUCUCCCCAU
sgRNA23_ Target 2	GGGGAGUCAGGCAACUAUGGAUGGCUAUAGUUCCUUGAAAAAGUUGCUAUAGUAAG GGCAACAGACCCGAGGCGUUGGGGAUCGCCUAGCCCGUUUUUACGGGCUCUCCCCAU
sgRNA20_ Target 3	GAAAUGUGCGCGGAACCCCUGCUAUAGUUCCUUGAAAAAGUUGCUAUAGUAAGGGC AACAGACCCGAGGCGUUGGGGAUCGCCUAGCCCGUUUUUACGGGCUCUCCCCAU
sgRNA21_ Target 3	GGAAAUGUGCGCGGAACCCCUGCUAUAGUUCCUUGAAAAAGUUGCUAUAGUAAGGG CAACAGACCCGAGGCGUUGGGGAUCGCCUAGCCCGUUUUUACGGGCUCUCCCCAU
sgRNA22_ Target 3	GGGAAAUGUGCGCGGAACCCCUGCUAUAGUUCCUUGAAAAAGUUGCUAUAGUAAGG GCAACAGACCCGAGGCGUUGGGGAUCGCCUAGCCCGUUUUUACGGGCUCUCCCCAU
sgRNA23_ Target 3	GGGGAAAUGUGCGCGGAACCCCUGCUAUAGUUCCUUGAAAAAGUUGCUAUAGUAAG GGCAACAGACCCGAGGCGUUGGGGAUCGCCUAGCCCGUUUUUACGGGCUCUCCCCAU
Target DNA	AGCGCCCAATACGCAAACCGCCTCTCCCCGCGCGTTGGCCGATTCATTAATGCAGCTGG CACGACAGGTTTCCCGACTGGAAAGCGGGCAGTGAGCGCAACGCAATTAATGTGAGTT AGCTCACTCATTAGGCACCCCAGGCTTTACACTTTATGCTTCCGGCTCGTATGTTGTGTG GAATTGTGAGCGGATAACAATTTCACACAGGAAACAGCTATGACCATGATTACGCCAA GCTTGCATGCCTGCAGGTCGACTCTAGAGGATCCCCGGGTACCGAGCTCGAATTCACTG GCCGTCGTTTTACAACGTCGTGACTGGGAAAACCCTGGCGTTACCCAACTTAATCGCCT TGCAGCACATCCCCCTTTCGCCAGCTGGCGTAATAGCGAAGAGGCCCGCACCGATCGCC CTTCCCAACAGTTGCGCAGCCTGAATGGCGAATGGCGCCTGATGCGGTATTTTCTCCTTA CGCATCTGTGCGGTATTTCACACCGCATACGTCAAAGCAACCATAGTACGCGCCCTGTA GCGGCGCATTAAGCGCGGCGGGTGTGGTGGTTACGCGCAGCGTGACCGCTACACTTGCC AGCGCCCTAGCGCCCGCTCCTTTCGCTTTCTTCCCTTCCTTTCTCGCCACGTTCGCCGGCT TTCCCCGTCAAGCTCTAAATCGGGGGCTCCCTTTAGGGTTCCGATTTAGTGCTTTACGGC ACCTCGACCCCAAAAAACTTGATTTGGGTGATGGTTCACGTAGTGGGCCATCGCCCTGA TAGACGGTTTTTCGCCCTTTGACGTTGGAGTCCACGTTCTTTAATAGTGGACTCTTGTTC CAAACTGGAACAACACTCAACCCTATCTCGGGCTATTCTTTTGATTTATAAGGGATTTTG CCGATTTCGGCCTATTGGTTAAAAAATGAGCTGATTTAACAAAAATTTAACGCGAATTT TAACAAAATATTAACGTTTACAATTTTATGGTGCACTCTCAGTACAATCTGCTCTGATGC CGCATAGTTAAGCCAGCCCCGACACCCGCCAACACCCGCTGACGCGCCCTGACGGGCTT GTCTGCTCCCGGCATCCGCTTACAGACAAGCTGTGACCGTCTCCGGGAGCTGCATGTGT CAGAGGTTTTCACCGTCATCACCGAAACGCGCGAGACGAAAGGGCCTCGTGATACGCCT ATTTTTATAGGTTAATGTCATGATAATAATGGTTTCTTAGACGTCAGGTGGCACTTTTCG GGGAAATGTGCGCGGAACCCCTTTTCCGAAATTTGTTTATTTTTCTAAATACGGGGGGAA ATTAGGTGCGCTTGGCTTTCCCAATGTATCCGCTCATGAGACAATAACCCTGATAAATGC TTCAATAATATTGAAAAAGGAAGAGTATGAGTATTCAACATTTCCGTGTCGCCCTTATTC CCTTTTTTGCGGCATTTTGCCTTCCTGTTTTTGCTCACCCAGAAACGCTGGTGAAAGTAA AAGATGCTGAAGATCAGTTGGGTGCACGAGTGGGTTACATCGAACTGGATCTCAACAGC GGTAAGATCCTTGAGAGTTTTCGCCCCGAAGAACGTTTTCCAATGATGAGCACTTTTAAA

Target DNA	GTTCTGCTATGTGGCGCGGTATTATCCCGTATTGACGCCGGGCAAGAGCAACTCGGTCG<br>CCGCATACACTATTCTCAGAATGACTTGGTTGAGTACTCACCAGTCACAGAAAAGCATC<br>TTACGGATGGCATGACAGTAAGAGAATTATGCAGTGCTGCCATAACCATGAGTGATAAC<br>ACTGCGGCCAACTTACTTCTGACAACGATCGGAGGACCGAAGGAGCTAACCGCTTTTTT<br>GCACAACATGGGGGATCATGTAACTCGCCTTGATCGTTGGGAACCGGAGCTGAATGAAG<br>CCATACCAAACGACGAGCGTGACACCACGATGCCTGTAGCAATGGCAACAACGTTGCG<br>CAAACTATTAACTGGCGAACTACTTACTCTAGCTTCCCGGCAACAATTAATAGACTGGA<br>TGGAGGCGGATAAAGTTGCAGGACCACTTCTGCGCTCGGCCCTTCCGGCTGGCTGGTTT<br>ATTGCTGATAAATCTGGAGCCGGTGAGCGTGGGTCTCGCGGTATCATTGCAGCACTGGG<br>GCCAGATGGTAAGCCCTCCCGTATCGTAGTTATCTACACGACGGGGAGTCAGGCAACTA<br>TGGATGTTTCCGAACGAAATAGACAGATCGCTGAGATAGGTGCCTCACTGATTAAGCAT<br>TGGTAACTGTCAGACCAAGTTTACTCATATATACTTTAGATTGATTTAAAACTTCATTTT<br>TAATTTAAAAGGATCTAGGTGAAGATCCTTTTTGATAATCTCATGACCAAAATCCCTTAA<br>CGTGAGTTTTCGTTCCACTGAGCGTCAGACCCCGTAGAAAAGATCAAAGGATCTTCTTG<br>AGATCCTTTTTTTCTGCGCGTAATCTGCTGCTTGCAAACAAAAAAACCACCGCTACCAG<br>CGGTGGTTTGTTTGCCGGATCAAGAGCTACCAACTCTTTTTCCGAAGGTAACTGGCTTCA<br>GCAGAGCGCAGATACCAAATACTGTCCTTCTAGTGTAGCCGTAGTTAGGCCACCACTTC<br>AAGAACTCTGTAGCACCGCCTACATACCTCGCTCTGCTAATCCTGTTACCAGTGGCTGCT<br>GCCAGTGGCGATAAGTCGTGTCTTACCGGGTTGGACTCAAGACGATAGTTACCGGATAA<br>GGCGCAGCGGTCGGGCTGAACGGGGGGTTCGTGCACACAGCCCAGCTTGGAGCGAACG<br>ACCTACACCGAACTGAGATACCTACAGCGTGAGCTATGAGAAAGCGCCACGCTTCCCGA<br>AGGGAGAAAGGCGGACAGGTATCCGGTAAGCGGCAGGGTCGGAACAGGAGAGCGCAC<br>GAGGGAGCTTCCAGGGGGAAACGCCTGGTATCTTTATAGTCCTGTCGGGTTTCGCCACC<br>TCTGACTTGAGCGTCGATTTTTGTGATGCTCGTCAGGGGGGCGGAGCCTATGGAAAAAC<br>GCCAGCAACGCGGCCTTTTTACGGTTCCTGGCCTTTTGCTGGCCTTTTGCTCACATGTTCT<br>TTCCTGCGTTATCCCCTGATTCTGTGGATAACCGTATTACCGCCTTTGAGTGAGCTGATA<br>CCGCTCGCCGCAGCCGAACGACCGAGCGCAGCGAGTCAGTGAGCGAGGAAGCGGAAG

The target sequence and the PAM sequence are colored red and purple, respectively.

Supplementary Table 2. sgRNA and target DNA sequence used for crystallization.

Name	Sequence
sgRNA	GGAAAUUAGGUGCGCUUGGCGCUAUAGUUCCUUGAAAAAGUUGCUAUAGUAAGGG CAACAGACCCGAGGCGUUGGGGAUCGCCUAGCCCGUUUUUACGGGCUCUCCCCAU
Target strand	CCTTTAATCCACGCGAACCGAAAGGTTT
Non-target strand	TTTCCAAA

The target sequence and the PAM sequence are colored red and purple, respectively.

Supplementary Table 3. Plasmids used for *in vivo* editing experiments

Name	Sequence
pSI-545-BlCas9	https://benchling.com/s/seq-cFTEo6rGaFhqDZfXSLsN?m=slm-wbUvTh9lDt2Eo0yMJAlI
pSI-545-RA	https://benchling.com/s/seq-pe3sJeepSoU6UgXl1Qn6?m=slm-BBLPiwfJSWn8vNLT4XeO
pSI-545-BlCas9-AID	https://benchling.com/s/seq-IIUsGUG2mTg6qxpOv61z?m=slm-s5mMuOJNXQ4N7acQ5HM6
pSI-545-RA-AID	https://benchling.com/s/seq-wgJr7A0CVPuMYyEpbA95?m=slm-JxMQf7UfTuNQwaR0OELS
pSI-414-BlCas9-sgRNA	https://benchling.com/s/seq-mDQV9quxozuz044hFKyc?m=slm-4KdqdbicHQhwI4Zxg8ZS

Supplementary Table 4. Target sequences for genome- and base-editing analyses.

Gene	Sequence	PAM
DYRK1A	GCCCACCAGGTCCTCCTGTTTC	N_4CNAA
MECP2	GCTTGCTCGTATTCATTCAAAA	N_4CNAA
PTEN	GACTACAAGCCTCACTTCATTC	N_4CNAA
DYRK1A	GAGGTCTTTGAACTTCAAGTAG	N_4CNAC
MECP2	GACCAAAATAAACATCTTCTCA	N_4CNAC
PTEN	GGGCATTAAAATATATGCATTA	N_4CNAC
DYRK1A	GTAGGCCCTAGGGCAAGGTGGA	N_4CNAG
MECP2	GCTTGAGGGACTAAATGTCACC	N_4CNAG
PTEN	GATTCATGAAAGCCAAGACTTT	N_4CNAG
DYRK1A	GTGATCTAAAGTTAGAATAAGT	N_4CNAT
MECP2	GTGCTTGTCGGTAAGAAAAACA	N_4CNAT
PTEN	GAAGCCTGTCCCGCACTTCTTA	N_4CNAT
DYRK1A	GTCCGGACTAGATCTTACAATC	N_4CNCA
MECP2	GCTTCGTGGGAACCCTATGTGC	N_4CNCA
PTEN	GAGGCTTCCTTCCCTTAAGCGG	N_4CNCA
DYRK1A	GCAATTCTACTCTTAGACTGTG	N_4CNGA
MECP2	GTCACCAATTCAAGCCAGTTTG	N_4CNGA
PTEN	GAACTTGTCTTCCCGTCGTGTG	N_4CNGA
DYRK1A	GACCACTGACCCGGGCGGAACA	N_4CNTA
MECP2	GGCCAGACACAAAAGGCCAAAT	N_4CNTA
PTEN	GGTCACATACCAGAGATATGTG	N_4CNTA
